# Identification of Estrogen Response Element in Aquaporin-3 Gene that Mediates Estrogen-induced Cell Migration and Invasion in Estrogen Receptor-positive Breast Cancer

**DOI:** 10.1038/srep12484

**Published:** 2015-07-29

**Authors:** Yi-Ting Huang, Jun Zhou, Shuai Shi, Hai-Yan Xu, Fan Qu, Dan Zhang, Yi-Ding Chen, Jing Yang, He-Feng Huang, Jian-Zhong Sheng

**Affiliations:** 1 Department of Pathology and Pathophysiology, School of Medicine, Zhejiang University, Hangzhou, Zhejiang, China; 2 The Key Laboratory of Reproductive Genetics, Ministry of Education (Zhejiang University), Hangzhou, Zhejiang, China; 3 Women’s Hospital, School of Medicine, Zhejiang University, Hangzhou, Zhejiang, China; 4 Department of Medical Reproductive Centre, People’s Hospital of Jinhuan City, Jinhua, Zhejiang, China; 5 The Second Affiliated Hospital, School of Medicine, Zhejiang University, Hangzhou, Zhejiang, China; 6 International Peace Maternity and Child Health Hospital, School of Medicine, Shanghai Jiao Tong University, Shanghai, China

## Abstract

Accumulating evidence suggests that aquaporins (AQPs) may facilitate tumor development. The molecular pathways connecting the pathological functions of AQPs are unclear and need to be better defined. This study aimed to investigate whether AQP3, one of the AQPs expressed highly in breast cancer, had any clinical implication in estrogen-receptor (ER) positive breast cancer, and explore the regulatory mechanisms of AQP3 in estrogen-related breast cancer progression. Here we show that AQP3 is an important enforcer of migration and invasion in breast cancer. We, for the first time, reported that ER-positive breast cancer tissues obtained from premenopausal patients had higher AQP3 expression when compared to those obtained from postmenopausal patients. Estrogen directly upregulates AQP3 by activating ERE in the promoter of the *AQP3* gene. The upregulation of AQP3 can influence the expression of molecules related to epithelial-mesenchymal transition and the reorganization of actin-cytoskeleton, resulting in enhancement of cell migration and invasion in ER-positive breast cancer cells.

Breast cancer is the most common cancer in women worldwide. The majority of breast cancers are estrogen-dependent for tumor progression^1^. Well understanding of the mechanisms of cell migration, invasion and proliferation in breast cancer is important for investigating possible anti-tumor therapies.

Aquaporins (AQPs) are a class of small integral membrane proteins distributed widely in organisms^2^,^3^. Thirteen members (AQP0-12) have been identified in mammals. AQP3, a member of the aquaglyceroporin subgroup, has broad tissue distribution in human body including renal collecting duct, epidermis, conjunctiva and mammary glands^4^,^5^. It has been reported that AQP3 could facilitate cell migration by transportation of water and glycerol for lamellipodia formation^6^, and lead to cellular proliferation by maintaining a high level of cellular glycerol used for the generation of ATP and lipid biosynthesis^7^. Mice lacking AQP3 showed defects in urinary-concentrating function^8^, skin wound healing^6^ and alimentary tract repairing^9^. Conversely, enhancement of AQP3 function, by upregulating AQP3 expression, may promote tumorigenesis and tumor development^7^,^10^,^11^.

Recent studies showed that several kinds of tumors including breast cancer overexpressed AQP3^12^,^13^,^14^,^15^,^16^,^17^,^18^. However, whether high expression level of AQP3 in breast cancer has any clinical implication in patients is poorly understood. On the other hand, the mechanisms underlying AQP3 upregulation in breast cancer also remain unclear. Because estrogen has been shown to be an important determinant of the risk of breast cancer^1^,^19^, we firstly investigated the relationship between the expression level of AQP3 in estrogen receptor (ER)-positive breast cancer and the patient characteristics. We then examined whether estrogen could alter the expression level of AQP3 in breast cancer cell lines. Finally, we successfully identified an estrogen response element (ERE) in the promoter of *AQP3* gene, which might mediate estrogen-induced AQP3 expression, cell migration and invasion in ER-positive breast cancer.

## Results

### Immunochistochemical analysis of AQP3 expression in the cancer tissues of patients with ER-positive breast cancer.

**Figure 1:**
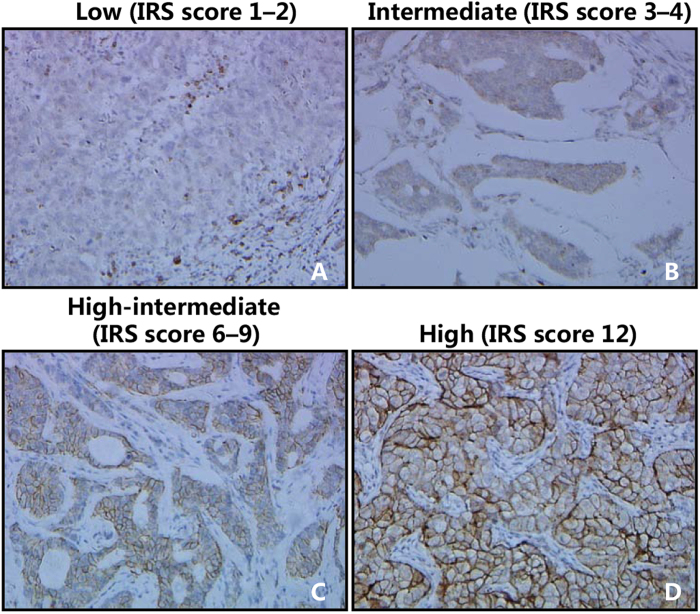
Immunochistochemical analysis of AQP3 expression in cancer tissues of patients with breast cancer. AQP3 IRS scores of representative examples of low (**A**), intermediate (**B**), high-intermediate (**C**), and high (**D**) are shown in ER-positive breast cancer, respectively (magnification: ×200). The tumor status (histopathological grade, nodal status, stage) of the representative examples is detailed as follows, (**A**) (1, N, 1), (**B**) (2, N, 1), (**C**) (3, P, 2) and (**D**) (3, P, 3). P: positive nodal metastasis; N: negative nodal metastasis.

Using immunohistochemistry (IHC) and immunoreactivity scoring system (IRS), we examined the expression level of AQP3 protein in breast invasive ductal carcinoma samples obtained from 56 patients. Before the IHC experiments, the AQP3 antibody had been proofed appropriately validated for IHC (Supplementary Figure S1A). Fig. 1 shows different IRS scores in breast cancer samples. We found that AQP3 was mainly expressed in the cell membrane and cytoplasm (Fig. 1 and Supplementary Figure S1B). The IRS analysis showed that higher AQP3 expression level was associated with higher histopathological grade and more lymph node metastasis in the patients with ER-positive breast cancer (Table 1). On the other hand, AQP3 expression level in ER-positive breast cancer was higher in the premenopausal patients than which in the postmenopausal patients (Table 1).

### Estrogen upregulated AQP3 expression in the ER-positive breast cancer cells

**Figure 2:**
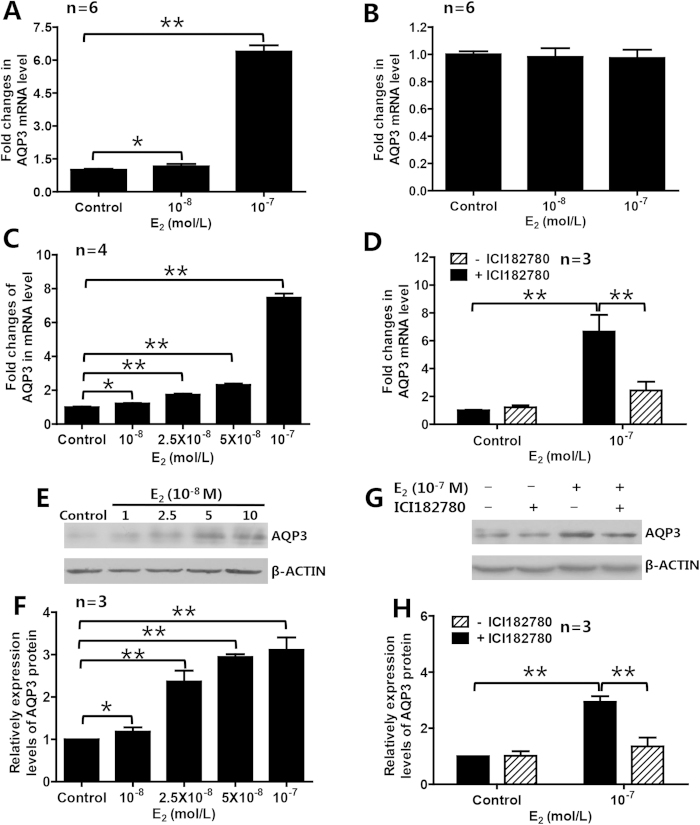
E_2_ upregulated AQP3 expression in ER-positive breast cancer cells. E_2_ upregulated expression of AQP3 in ER-positive T47D cells (**A**), but not in ER-negative MDA-MB-231 cells (**B**). In T47D cells, E_2_ dose-dependently increased AQP3 mRNA (**C**) and protein (**E**,**F**) expression. ER antagonist ICI182780 could block the E_2_-induced expression of AQP3 mRNA (**D**) and protein (**G**,**H**). The data are presented as means ± SD, *P < 0.05 and **P < 0.01 (**A**–**C**,**F** One-way ANOVA and Turkey’s post hoc tests; **D**,**H** Student *t*-test).

In order to determine whether and how estrogen regulates AQP3 expression in ER-positive breast cancer cells, we treated three breast cancer cell lines including ER-positive T47D and MCF7 cells and ER-negative MDA-MB-231 cells with estradiol (E_2_), and found that treatment with 10^−8^ M and 10^−7^ M E_2_ for 48 h significantly upregulated the expression level of AQP3 mRNA in ER-positive breast cancer cells (T47D, Fig. 2A; MCF7, Supplementary Figure S2A), but not in ER-negative breast cancer cells (MDA-MB-231, Fig. 2B). The E_2_-induced upregulation of AQP3 mRNA and protein expression in T47D cells was dose-dependent (Fig. 2C,E,F). The estrogenic effects on AQP3 mRNA and protein expression in T47D cells were blocked by 10^−6^ M ICI182780, an estrogen receptor antagonist^20^, suggesting that estrogen receptors may mediate the estrogen-induced upregulation of AQP3 in ER-positive breast cancer cells (Fig. 2D,G,H).

### Identification of a functional ERE in the promoter of *AQP3* gene

**Figure 3:**
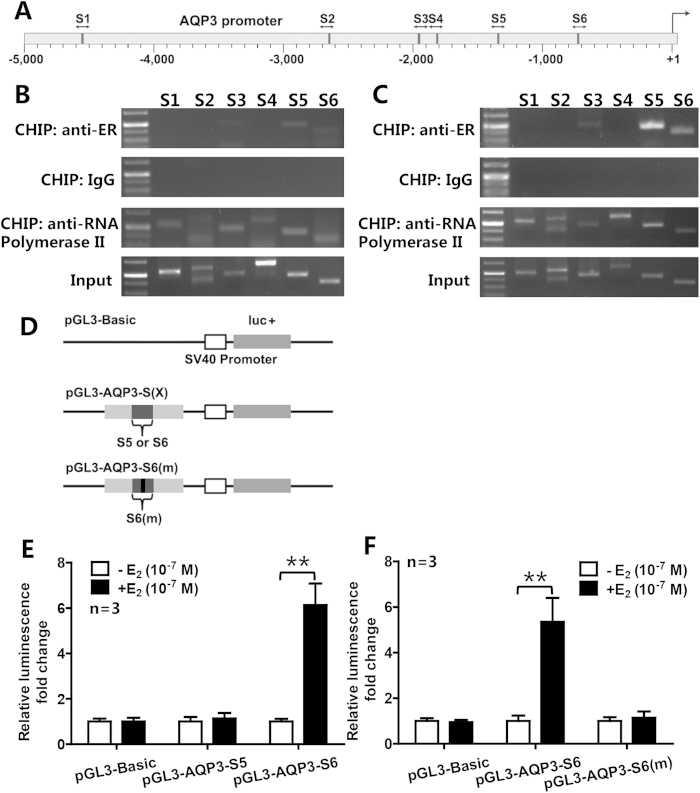
Identification of the functional ERE in the promoter of *AQP3* gene. (**A**) Schematic depiction of the *AQP3* promoter suggested six sequences (S1-S6, dark grey rectangles) which might contain putative EREs. (**B**,**C**) ChIP analysis included positive control (anti-RNA polymerase II), negative control (normal mouse IgG), ERα antibody and input groups. Three sequences (S3, S5 and S6) were pulled down by anti-ERα antibody, and were brighter in the presence of E_2_ (**C**) than in the absence of E_2_ (**B**). After sequencing, S3 was excluded. (**D**) Schematic of *AQP3* promoter-driven luciferase reporter constructs was indicated. PGL3-Basic was used as a negative control. pGL3-AQP3-S5 and pGL3-AQP3-S6 contained S5 and S6, respectively. AQP3-luc-S6(m) contained a mutated S6, and the mutation site was in its putative ERE. (**E**,**F**) Luciferase activities of the report systems showed that only pGL3-AQP3-S6 could be activated by E_2_, and E_2_ had no effect on the mutated pGL3-AQP3-S6(m). The data are presented as mean ± SD, **P < 0.01 (Student *t*-test).

In order to determine whether the *AQP3* gene in ER-positive breast cancer cells is regulated directly by estrogen via ER binding to ERE, we analyzed putative EREs in promoter of *AQP3* gene using the Regulatory Sequence Analysis Tools (RSAT), and, obtained six high-score putative EREs (Fig. 3A). ChIP analysis showed that three fragments (S3, S5 and S6) in promoter of *AQP3* could be pulled down by ERα antibody (Fig. 3B,C; Supplementary Figure S2B and S2C), and were brighter in the presence of E_2_ (Fig. 3C and Supplementary Figure S2C). After sequencing, S3 was excluded. To determine whether the pulled down fragments, including S5 and S6, had a functional role in estrogen-dependent transcriptional activation, we constructed two plasmids (pGL3-AQP3-S5 and pGL3-AQP3-S6, Fig. 3D) for luciferase reporter assay. The results showed that pGL3-AQP3-S6 that contained a putative ERE (ACATGGCTaggTGACCTAG) was activated by E_2_ (Fig. 3E), whereas E_2_ had no effect on pGL3-AQP3-S5 and the mutated pGL3-AQP3-S6(m) (ACATGGCTaggCCTAG) (Fig. 3E,F). This result indicates that the promoter of *AQP3* gene contains a functional ERE motif, which may mediate estrogen-induced upregulation of AQP3 expression in ER-positive breast cancer cells.

### Knockdown or inhibition of AQP3 significantly reduced the E_2_-induced migration and invasion of ER-positive breast cancer cells

**Figure 4:**
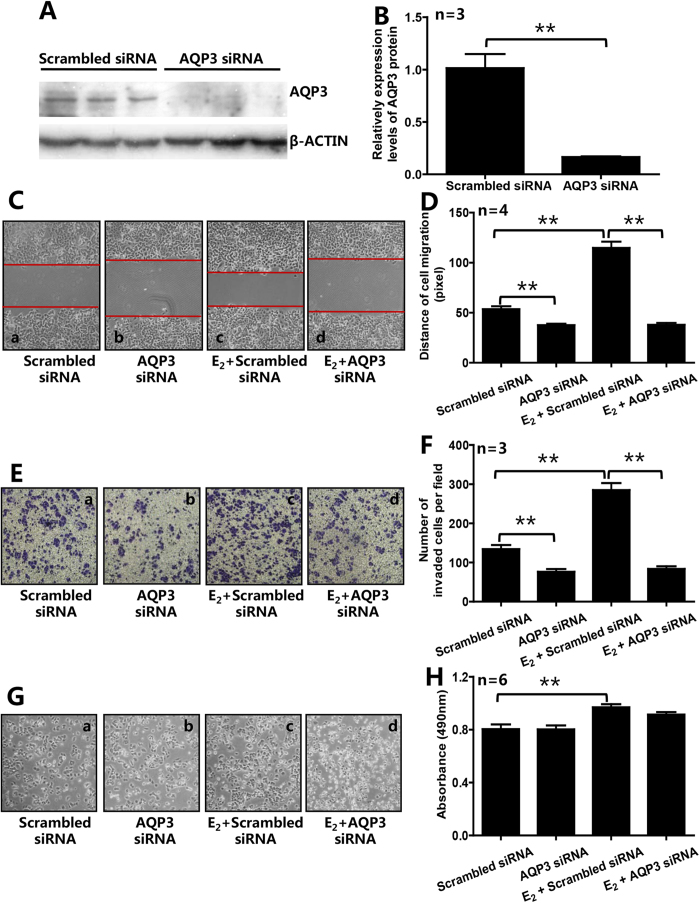
Knockdown of AQP3 reduced E_2_-induced cell migration and invasion of ER-positive breast cancer cells. (**A**,**B**) Treating T47D cells with AQP3-specific siRNA significantly reduced the expression level of AQP3. Knockdown of AQP3 attenuated migration (**C**,**D**) and invasion (**E**,**F**) of T47D cells in the absence of E_2_. E_2_ (10^−7^ M) significantly increased cell migration (**C**,**D**), invasion (**E**,**F**), and proliferation (**G**,**H**) in scrambled siRNA group, and, Knockdown of AQP3 in T47D cells inhibited the E_2_-promoted migration and invasion, but not proliferation (**C–H**). The data are presented as mean ± SD, **P < 0.01 (**B** Student *t*-test; **D**,**F** and **H** One-way ANOVA and Turkey’s post hoc tests).

In order to determine the role of AQP3 in E_2_-induced migration, invasion and proliferation of ER-positive breast cancer cells, we transfected siRNA targeting *AQP3* gene into T47D and MCF7 cells. Compared with the cells transfected with scrambled siRNA, treating T47D cells with AQP3 siRNA for 36 h significantly reduced the expression level of AQP3 protein by 84.9% (Fig. 4A,B). E_2_ (10^−7^ M) significantly promoted cell migration (2.14 ± 0.23-flod), invasion (2.12 ± 0.23-flod) and proliferation (1.21 ± 0.07-flod) in T47D cells transfected with scrambled siRNA (Fig. 4C–H). As shown in Fig. 4C,D, knockdown of AQP3 decreased 29.7% of the migration distance in AQP3 siRNA group (vs. scrambled siRNA group), and 66.9% in AQP3 siRNA + E_2_ group (vs. scrambled siRNA + E_2_ group). As shown in Fig. 4E,F, knockdown of AQP3 significantly reduced 43.2% of the invaded cells in AQP3 siRNA group (vs. scrambled siRNA group), and 70.6% in AQP3 siRNA + E_2_ group (vs. scrambled siRNA + E_2_ group). However, knockdown of AQP3 had no effect on cell proliferation (Fig. 4G,H). We also observed the similar results in another ER-positive breast cancer cell line MCF7 (Supplementary Figure S4). Treating MCF7 cells with E_2_ (10^−7^ M) significantly promoted cell migration (Supplementary Figure S4C and S4D) and invasion (Supplementary Figure S4E and S4F). As shown in Supplementary Fig. S4C and S4D, knockdown of AQP3 significantly decreased 39.2% of the migration distance in AQP3 siRNA group (vs. scrambled siRNA group), and 56.9% in AQP3 siRNA + E_2_ group (vs. scrambled siRNA + E_2_ group). As shown in Supplementary Fig. S4E and S4F, knockdown of AQP3 significantly reduced 55.1% of the invaded cells in AQP3 siRNA group (vs. scrambled siRNA group), and 66.5% in AQP3 siRNA + E_2_ group (vs. scrambled siRNA + E_2_ group).

Pretreatment of CuSO_4_, an inhibitor of AQP function, also significantly reduced the E_2_-induced migration and invasion in T47D cells (Supplementary Figure S3). As shown in Supplementary Fig. S3A and S3B, CuSO_4_ significantly decreased 41.2% of the migration distance in CuSO_4_ group (vs. control group), and 66.8% in CuSO_4_ + E_2_ group (vs. E_2_ group). As shown in Supplementary Fig. S3C and S3D, CuSO_4_ significantly reduced 40.6% of the invaded cells in CuSO_4_ group (vs. control group), and 65.1% in CuSO_4_ + E_2_ group (vs. E_2_ group).

### AQP3 overexpression enhanced the migration and invasion of T47D cells

**Figure 5:**
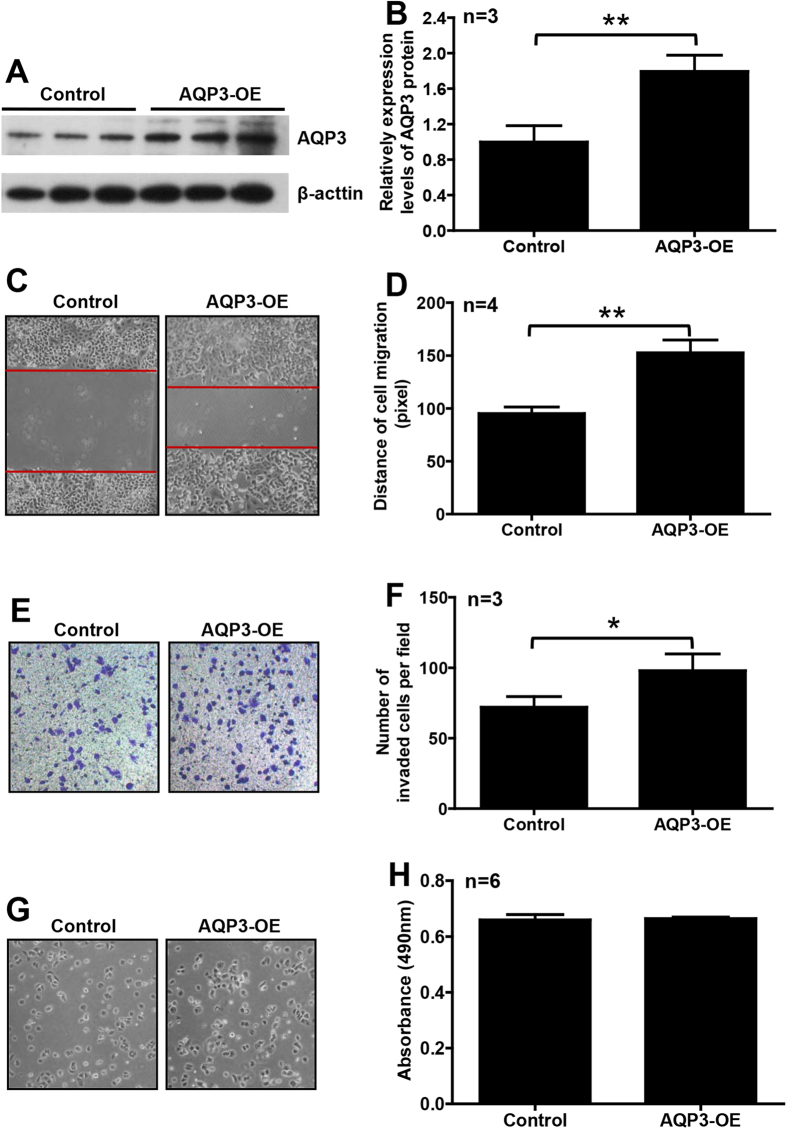
Overexpression of AQP3 enhanced migration and invasion of ER-positive breast cancer cells. The expression level of AQP3 was significantly increased in T47D cells with vector-mediated overexpression (AQP3-OE) (**A**,**B**). Overexpression of AQP3 significantly enhanced migration (**C**,**D**) and invasion (**E**,**F**), but not proliferation (**G**,**H**), of T47D cells. The data are presented as mean ± SD, *P < 0.05 and **P < 0.01 (Student *t*-test).

To confirm that high AQP3 expression might play an important role in breast cancer progression, we overexpressed AQP3 in T47D cells. After AQP3 overexpression vector transfection for 48 h, AQP3 protein level was significantly increased (161.9%), compared with control (Fig. 5A,B). As shown in Fig. 5C–H, the cells overexpressing AQP3 showed significantly increased migration (1.60 ± 0.13-fold) and invasion (1.36 ± 0.16-fold), not proliferation (1.01 ± 0.02-fold).

### Knockdown or inhibition of AQP3 significantly reduced the E_2_-induced reorganization of actin cytoskeleton in T47D cells.

**Figure 6:**
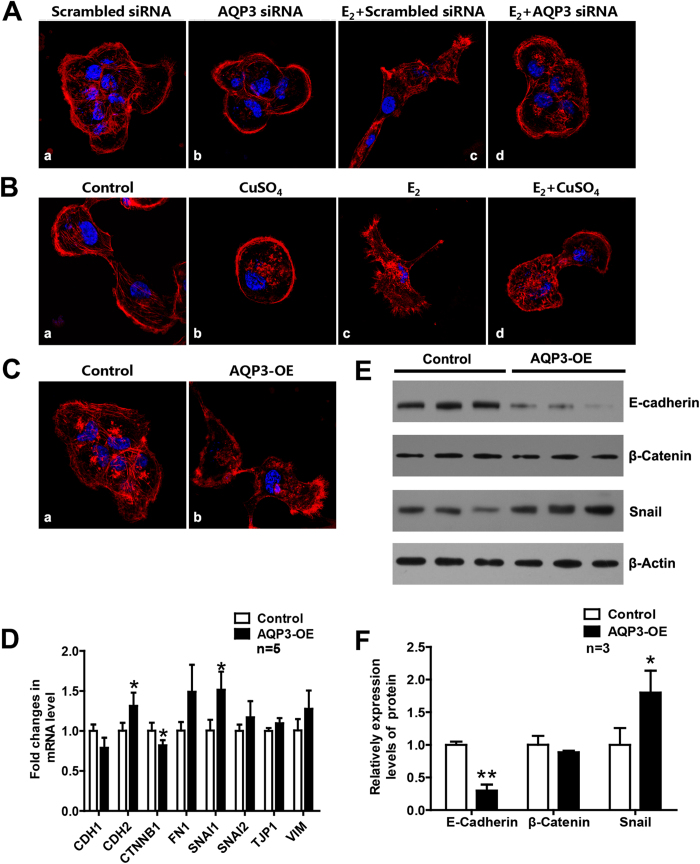
AQP3 influenced reorganization of actin cytoskeleton and expression of EMT-related factors. (**A**,**B**) Organization of the actin cytoskeleton in T47D cells treated with or without AQP3 siRNA or CuSO_4_, respectively. (**Ac** and **Bc**) E_2_ induced the reorganization of actin cytoskeleton (red) by promoting formation of filopodia and rearrangement of stress fibers. Treating T47D cells with AQP3 siRNA (**Ab** and **Ad**) or CuSO_4_ (**Bb** and **Bd**) significantly reduced E_2_-regulated reorganization of actin cytoskeleton. (**C**) AQP3 overexpression influence reorganization of actin cytoskeleton. (**D–F**) The expression of several MET-related factors changed after AQP3 overexpressed. The data are presented as mean ± SD, *P < 0.05 and **P < 0.01 (Student *t*-test).

In T47D cells, E_2_ induced a marked reorganization of actin cytoskeleton characterized by formation of filopodia and rearrangement of stress fibers (Fig. 6Ac,Bc), which contributed to the E_2_-induced migration and invasion. Knockdown of AQP3 (Fig. 6Ab,Ad) or inhibition of AQP3 function with CuSO_4_ (Fig. 6Bb,Bd) significantly reduced E_2_-regulated reorganization of actin cytoskeleton.

### AQP3 overexpression influenced reorganization of actin cytoskeleton and expression of EMT-related factors in T47D cells.

AQP3 overexpression induced reorganization of actin cytoskeleton in T47D cells, which characterized by formation of filopodia and rearrangement of stress fibers (Fig. 6Cb). To explore the mechanism underlying migration and invasion promoted by AQP3, we analyzed the expression of several molecules related to epithelial-mesenchymal transition (EMT), including the epithelial markers *CDH1* (E-cadherin), *CTNNB1* (β-catenin) and *TJP1* (ZO-1), and, the mesenchymal markers *CDH2* (N-cadherin), *VIM* (Vimentin), *FN1* (Fibronectin 1), *SNAI1* (Snail) and *SNAI2* (Slug), between control and AQP3-overexpressed T47D cells by qPCR. As shown in Fig. 6D, the mRNA level of one factor (*CTNNB1*) was decreased and two (*CDH2* and *SNAI1*) were increased significantly (*P* < 0.05) in AQP3-overexpression group. The results were further verified by Western blotting analysis (Fig. 6E,F), suggesting that AQP3 might act as a positive regulator of the EMT signaling pathways during the metastasis of breast cancer, although the mechanisms was yet unknown.

## Discussion

The present study showed that higher AQP3 expression level in ER-positive breast cancer tissues was associated with higher histopathological grade and more lymph node metastasis. On the other hand, we found that AQP3 expression level was higher in samples obtained from premenopausal patients than those from postmenopausal patients. We identified an ERE in the promoter of *AQP3* gene, and found that estrogen could promote cell migration and invasion in ER-positive breast cancer via activating the ERE in *AQP3* gene. Knockdown of AQP3 significantly attenuated migration and invasion of breast cancer cells, and over-expression of AQP3 significantly enhanced these two processes. AQP3 mediated E_2_-enhanced migration and invasion through influencing the expression of EMT-related factors and the reorganization of actin cytoskeleton.

Several studies showed that some subtypes of aquaporins including AQP1, 3 and 5 were elevated in breast cancer tissues compared to normal tissues^17^,^21^,^22^. In clinical studies, it has been suggested that high AQP1 expression might be associated with poor prognosis^22^, and high AQP5 expression might be associated with more lymph node invasion^17^,^21^. In this study, we explored the relationship between AQP3 expression level in ER-positive breast cancer and the clinical characteristics of patients with breast invasive ductal carcinoma, by immunohistochemistry. Immunoreactivity analysis showed that ER-positive breast cancer tissues obtained from premenopausal patients had higher AQP3 level than those obtained from postmenopausal patients, and treating ER-positive breast cancer cells with E_2_ not only upregulated the expression of AQP3, but also promoted cell migration and invasion. The results suggest that AQP3 may mediate estrogen-promoted tumor development in ER-positive breast cancer.

The mechanisms underlying the regulation of AQP3 expression are complex and unclear. It was reported that natriuretic peptides could upregulate AQP3 in human colonic epithelial cells^23^, and, TNF-α could upregulate AQP3 in the epithelial lesion with chronic periodontitis^24^. Further studies demonstrated that growth factors like EGF and FGF-2 could upregulate AQP3 expression though MAPK/ERK pathway and/or PI3K/AKT pathway^11^,^16^,^25^. However, the regulatory mechanisms for AQP3 expression in breast cancer were still poorly understood. Previous researches showed that, in rat model, the expression of AQP3 in urothelium^26^ and vagina^27^ was significantly lower after ovariectomy and could be restored to the control levels after estrogen treatment. Since estrogen is critical for development and progression of breast cancer, we investigated whether E_2_ could enhance AQP3 expression in ER-positive breast cancer cell line (T47D) or ER-negative breast cancer cell line (MDA-MB-231). Our data showed that E_2_ could upregulate AQP3 expression in ER-positive cells only, and the ER antagonist inhibited its enhanced effects. Estrogen exerts its effects by directly binding to ER, which homodimerizes and interacts with ERE in genes to stimulate the transcription of target genes. We, for the first time, found a functional ERE in the promoter region of the *AQP3* gene. Although this sequence is exactly same as the canonical ERE (GGTCAcagTGACC), the 13-nucleotide (TGGCTaggTGACC) can form an inverted repeat just like the classical ERE. We are now fully persuaded the expression of AQP3 is under the direct control of estrogen.

On the other hand, we found that higher AQP3 levels were associated with poorer cell differentiation and more lymph node metastasis in ER-positive breast cancer patients, suggesting that AQP3 may be an enhancer in breast cancer progression. It has been shown that AQP3 might mediate FGF-2-induced cell migration in two representative breast cancer cell lines (MDA-MB-231 and Bcap-37)^25^. This study demonstrated that AQP3 played an important role in E_2_-induced migration and invasion of ER-positive (T47D) breast cancer cells. Knockdown of AQP3 expression significantly attenuated migration and invasion of T47D cells, while overexpression of AQP3 promoted cell migration and invasion.

For metastasis to occur, tumor cells firstly detach from their tissue of origin, and then migrate and invade to another organ^28^. Previous studies indicated that AQP3 expression level might have relevance with molecules which were important in cell-cell and/or cell-matrix interactions^24^,^29^,^30^,^31^,^32^. Those Data showed a significant decrease in matrix metalloproteinases (MT1-MMP, MMP-2 and M MP-9)^31^ and cell adhesion molecules (ICAM-1^33^, integrinα5β1^29^ and E-cadherin^30^) after AQP3 knockdown, suggesting that these molecules may play roles in AQP3-mediated migration and invasion of breast cancer cells. Our data showed that, in breast cancer cells, increased expression of AQP3 would upregulate the expression of Snail, downregulate the expression of E-cadherin, and, influence the formation of filopodia and the rearrangement of stress fibers.

In conclusion, we used a combination of clinical patient samples and *in vitro* cellular systems to show that AQP3 plays an important role in cell migration and invasion of ER-positive breast cancer. Our clinical data indicated that higher AQP3 expression in ER-positive breast cancer was associated with poorer cell differentiation, more lymph node metastasis and premenopausal status. We, for the first time, identified an ERE in the promoter of *AQP3* gene, and found that estrogen might promote breast cancer development through activating ERE in the promoter of *AQP3* gene and upregulating AQP3 expression in ER-positive breast cancer. Upregulation of AQP3 by E_2_ increases the cell migration and invasion through regulating the expression of EMT-related factors and influencing the reorganization of actin cytoskeleton. Hence, in our view, AQP3 might be a potential target for anti-breast cancer treatment. However, the precise molecular mechanisms of AQP3-induced cell migration and invasion are still incompletely understood. Further studies are encouraged to investigate the mechanisms.

## Methods

### Patients and sample collection

This study included Fifty-six patients (median age, 50 yr; range: 31–69 yr) with breast invasive ductal carcinoma (IDC) confirmed by histopathological analysis after breast surgery at Women’s Hospital, School of Medicine, Zhejiang University, China, between January 2011 and June 2012. Histopathological grade was determined by modified Bloom-Richardson-Elston grading system. These patients did not receive hormone treatment in the previous three months. The study was approved by the Ethics Committee of Women’s Hospital, School of Medicine, Zhejiang University, and all patients provided the written informed consent for this study. All experiments were performed in accordance with relevant guidelines and regulations.

### Tissue immunohistochemistry

Breast samples were sectioned at 4 μm intervals. Sections were heated in citrate buffer for 20 min for antigen retrieval, and, bathed in a 3% H_2_O_2_/PBS solution for 10 min at room temperature in the dark to quench endogenous peroxidase. Then, sections were incubated with AQP3 primary antibody (Abcam, San Francisco, CA, USA) at a 1:500 dilution overnight at 4 °C. After several washes, 50 μl of secondary antibodies (DakoCytomation, Carpinteria, CA, USA) were added to tissues and incubated for 30 min. After washing again, the sections were reacted with DAB, and, counterstained with hematoxylin. Immunostained sections were reviewed and scored semiquantitatively by expert pathologists on the basis of a well-established immunoreactivity scoring system (IRS)^34^. The IRS score was calculated by the product of the percentage of positive cells (0, 0%; 1, 1–10%; 2, 11–50%; 3, 51–80%; 4, >80%) and the intensity of the staining (0, no staining; 1, mild; 2, moderate and 3, strong), which gave an IRS score from 0 (no staining) to 12 (maximum staining).

### Agar-paraffin double embedded technology

In order to validate the IHC results, the pellets of control, AQP3 knockdown and AQP3 overexpression T47D cells were embedded in the same paraffin block, and IHC was performed on the same panel. Cells were trypsinised and fixed for 3 h in 10% formalin, centrifuged at 2000 g for 10 minutes, then washed twice with PBS and stained with eosin, and finally re-suspended in 3% agarose. The cell pellets were processed through gradient concentrations of alcohols before being cleared in xylene and washed in molten paraffin. These cell pellets were embedded in paraffin and IHC was carried out on 4–5 μm sections.

### Cells and cell culture

Breast cancer cell lines, T47D and MDA-231 cells were obtained from the Key Laboratory of Cancer Prevention and Intervention, Ministry of Education, China, and, were cultured in RPMI-1640 and L-15, respectively, supplemented with 10% fetal bovine serum (FBS) and antibiotics. Before and during the E_2_ treatment, cells were cultured in phenol red-free medium supplemented with 10% charcoal/dextran-treated FBS.

### RT-qPCR

Total RNA was extracted from breast cancer cells using RNAiso Plus (Takara, Dalian, China) according to the manufacturer’s instructions. The cDNA was prepared by reverse transcription, using RT reagent Kit (Takara). qPCR was carried out with SYBR-Green premix Ex Taq (Takara) in an Applied Biosystems 7900 Fast (ABI, Carlsbad, CA, USA), using GAPDH as internal controls. Primer sequences used for qPCR are shown in Supplemental Table S2. qPCR was performed in a 10 μl reaction system containing 5 μl SYBR premix Ex Taq, 0.2 μl sense and 0.2 μl antisense primers, 0.2 μl Dye I, 3.4 μl ddH_2_O, 1.0 μl cDNA. The thermal cycling conditions were: 95 °C for 10 s, 95 °C for 5 s, 60 °C for 34 s, and for 40 cycles. Every sample was repeated at least three times. Data were analyzed by the comparative threshold cycle (CT) method.

### Protein extraction and western blot analysis

Protein extracts from breast cancer cell lines were made in RIPA buffer containing protease inhibitors (1 μg/mL leupeptin and 1 μg/mL phenylmethylsulfonyl fluoride). The polyvinylidene fluoride transfer membranes containing separated samples were incubated with blocking buffer for 30 min. The membranes were then incubated with primary antibody (AQP3, 1:500 or β-actin, 1:2000) overnight at 4 °C, followed by horseradish peroxidase-linked secondary antibody (1:5000) for 1 h at room temperature, and visualized with ECL detection reagent. The grayscale of bands was measured with Quantity One software.

### Bioinformation and chromatin immunoprecipitation (ChIP) analyses

The Regulatory Sequence Analysis Tools (RSAT) web server was used to analyze the promoter sequence of *AQP3* gene to find high-score putative EREs. T47D cells were treated with 10^−7^ M E_2_ for 6 h and cross-linked with 1% formaldehyde and processed. The ChIP analyses were performed according to the ChIP Kit Instruction Manual (Millipore, Billerica, MA, USA). Condition for sonication was 15 sec pulse followed by 30 sec rest, and power setting at 30%. ERα antibody (Millipore) is functionally validated in the precipitation of ERα associated chromatin. Purified immunoprecipitated DNA were used for RT- PCR and the PCR products were confirmed by sequencing. Primer sequences used for ChIP PCR are shown in Supplemental Table S1.

### Plasmid construction and luciferase reporter assay

*AQP3* promoter fragments containing an ERE-like sequence were gained from ChIP analyses. They were digested with *Xho*I and *Kpn*I, and ligated into promoter-less luciferase reporter plasmid pGL3-basic (Promega, Madison, WI, USA) to construct the *AQP3* promoter-luciferase reporter systems. Luciferase reporter systems (0.8 μg AQP3 promoter-luciferase reporter plasmid or pGL3-basic, and 0.08 μg pRL-TK reporter plasmid) were transfected into T47D cells in 24-well plate. After transfection for 36 h, cells were treated with or without E_2_ (10^−7^ M). Cell lysates were prepared, and, luciferase activities were measured using the dual-luciferase reporter assay system (Promega) according to the manufacturer’s instructions. Luciferase values were normalized to the Renilla luciferase activity.

### Interfering RNAs (siRNAs) knockdown and vector-mediated overexpression studies

T47D cells were seeded in 6-well plates. For knockdown experiments, siRNA targeting the *AQP3* gene (100 pmol/well) and siRNA negative control were purchased from RiboBio (Guangzhou, China). For overexpression experiments, AQP3 overexpression vector (2 μg/well) was purchased from OriGene (Rockville, MD, USA). Cell transfection was conducted using Lipofectamine 2000 (Invitrogen, Carlsbad, CA, USA) according to the manufacturer’s guideline.

### Wound healing assay

T47D cells (1 × 10^5^/well) were seeded in 12-well plates pre-coated with 0.5% gelatin overnight at 4 °C. After pretreatment (knockdown or overexpression of AQP3), cells were cultured to confluence overnight. The monolayer cells were then scratched with a standard 200 μl pipette tip, and washed twice with PBS to remove floating cells. After scratching the lines, cells were cultured in medium supplemented with or without E_2_ (10^−7^ M) for 24 h. Mitocycin C (10 mg/ml) was included in the medium to prevent cell proliferation. Wound healing was quantified by measuring the migratory distance of cells.

### Transwell invasion assay

A permeable filter of transwell system (Corning Incorporated, Midland, MI, USA) was used to study the invasion ability of cells. The inside compartment of the transwell inserts was coated with Matrigel (BD Biosciences, Bedford, MA, USA) at 4 °C overnight, and then blocked by 1% BSA/PBS solution for 30 min at room temperature. After pretreatment (knockdown or overexpression of AQP3), T47D cells (1 × 10^5^/well) were loaded in the upper chamber in culture medium with 0.2% BSA, and with or without E_2_ (10^−7^ M). Cell migration to the other side of the membrane was induced by 30% FBS-containing medium in the lower chamber for 24 h. Cells were fixed in methanol for 30 min, and stained with 0.5% crystal violet for 15 min. After gently removing the cells on the up side of the top chamber, migrated cells were photographed and counted with Image-J software (National Institutes of Health, Bethesda, MD, USA).

### Cell proliferation assay

T47D cells (1 × 10^4^/well) were plated in 96-well plates. After pretreatment (knockdown or overexpression of AQP3), cells were cultured for 24 h in culture medium supplemented with or without E_2_ (10^−7^ M). The MTT assay was applied to quantify cell proliferation, and, the absorbance of samples was measured at 490 nm.

### Immunofluorescence analysis

T47D cells (1 × 10^4^/well) were grown on coverslips and exposed to treatments. The fixed cells were blocked in 1% BSA and then incubated with a 1:300 dilution of primary AQP3 antibody at 4 °C overnight. They were then incubated with a 1:200 dilution of Alexa Fluor488 goat anti-rabbit IgG (Invitrogen, Carlsbad, CA, USA) for 1 h. Actin-cytoskeleton was detected using rhodamine-conjugated phalloidin (Sigma, St Louis, MO, USA) diluted in phosphate buffer (50 μg/mL). Confocal images were taken by Olympus microscope (BX61W1-FV1000).

### Statistical analysis

Statistical analysis was performed with two-tailed indirect Student’s *t*-test between two groups. One-way ANOVA and Turkey’s post hoc tests were used to evaluate the statistical significance of the difference between more than two groups. *P*-value < 0.05 was regarded as significant.

## Additional Information

**How to cite this article**: Huang, Y.-T. *et al.* Identification of Estrogen Response Element in Aquaporin-3 Gene that Mediates Estrogen-induced Cell Migration and Invasion in Estrogen Receptor-positive Breast Cancer. *Sci. Rep.* **5**, 12484; doi: 10.1038/srep12484 (2015).

## Supplementary Material

Supplementary Information

## Figures and Tables

**Table 1:** Patient characteristics and AQP3 expression in ER-positive.

Characteristics	ER-positive (n = 56)	
Age (years, mean (range))	50 (31–69)	
BMI (kg/m^2^)	23.06 ± 2.92	
E_2_ (pmol/L, mean (range))	286.86 (18.35–2322)	
	n (%)	AQP3 IRS score
Menses status		
Premenopausal	31 (55.4%)	6.25 ± 2.24
Postmenopausal	25 (44.6%)	5.05 ± 1.84*
Histopathological grade		
I-II	32 (57.1%)	4.72 ± 1.55
II-III	22 (39.3%)	7.28 ± 2.04**
Nodal status		
Negative	29 (51.8%)	5.10 ± 1.78
Positive	27 (48.2%)	6.24 ± 2.39*
Stage		
I-II	49 (87.5%)	5.38 ± 2.02
III	7 (12.5%)	8.03 ± 1.53**

Data are presented as mean ± SD, mean (range) or number (%). *P < 0.05, and **P < 0.01, compared with the corresponding controls, respectively.
