# Supplementary material for: Identification of Estrogen Response Element in Aquaporin-3 Gene that Mediates Estrogen-induced Cell Migration and Invasion in Estrogen Receptor-positive Breast Cancer
Source: Sci Rep. 2015 Jul 29;5:12484. doi: 10.1038/srep12484 (PMC4518221; doi:10.1038/srep12484)
Supplement: Supplementary Information [file srep12484-s1.doc]

**Supplementary Information**

**Identification of Estrogen Response Element in Aquaporin-3 Gene that Mediates Estrogen-induced Cell Migration and Invasion in Estrogen Receptor-positive Breast Cancer**

Yi-Ting Huang^1,2^, Jun Zhou^3^, Shuai Shi^4^, Hai-Yan Xu^2^, Fan Qu^3^, Dan Zhang^3^, Yi-Dind Chen^5^, Jing Yang^2^, He-Feng Huang^2,6^*, Jian-Zhong Sheng^1,2^*

^1^ Department of Pathology and Pathophysiology, School of Medicine, Zhejiang University, Hangzhou, Zhejiang, China

^2^ The Key Laboratory of Reproductive Genetics, Ministry of Education (Zhejiang University), Hangzhou, Zhejiang, China

^3^ Women’s Hospital, School of Medicine, Zhejiang University, Hangzhou, Zhejiang, China

^4^ Department of Medical Reproductive Centre, People’s Hospital of Jinhuan City, Jinhua, Zhejiang, China

^5^ The Second Affiliated Hospital, School of Medicine, Zhejiang University, Hangzhou, Zhejiang, China

^6^ International Peace Maternity and Child Health Hospital, School of Medicine, Shanghai Jiao Tong University, Shanghai, China

* Jian-Zhong Sheng (e-mail: shengjz@zju.edu.cn) and He-Feng Huang (e-mail: huanghefg@hotmail.com) are considered corresponding co-authors.


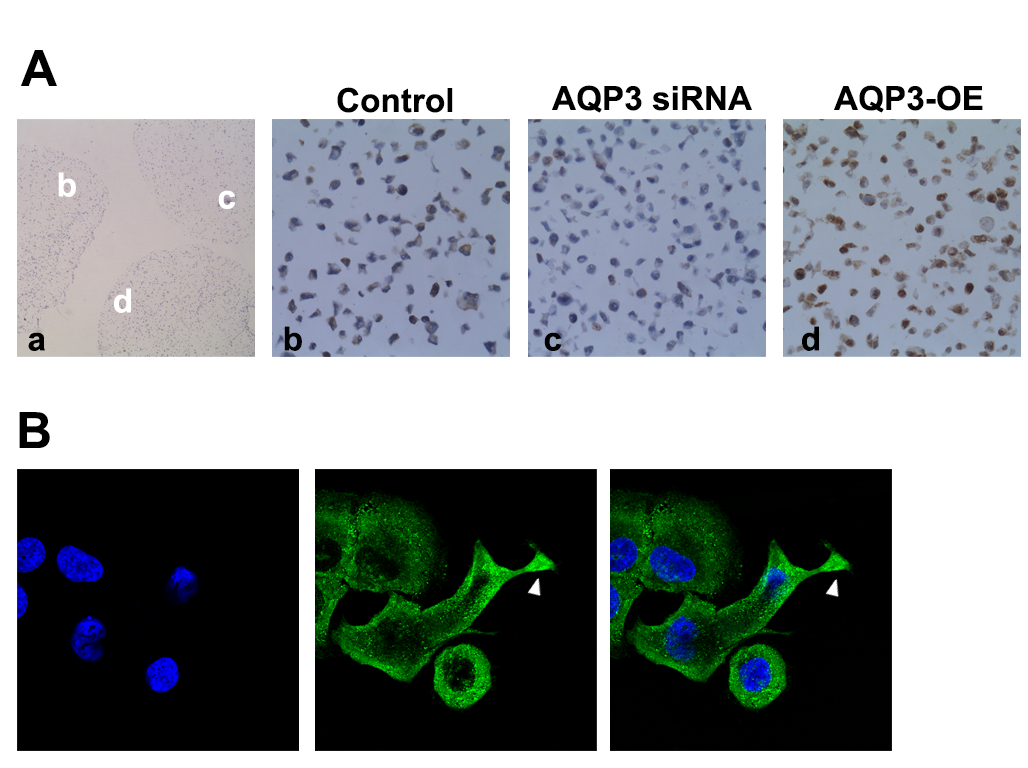


**Supplementary Figure S1:** **(A)** The pellets of T47D cells in control, AQP3 knockdown and AQP3 overexpression groups were embedded in the same paraffin block, and IHC of AQP3 was performed on the same pane (a, magnification: ×25; b-d, magnification: ×200). **(B)** Confocal image of AQP3 (green) in T47D cells (magnification: ×1200; blue: DAPI; white arrowhead: lamellipodia structures).


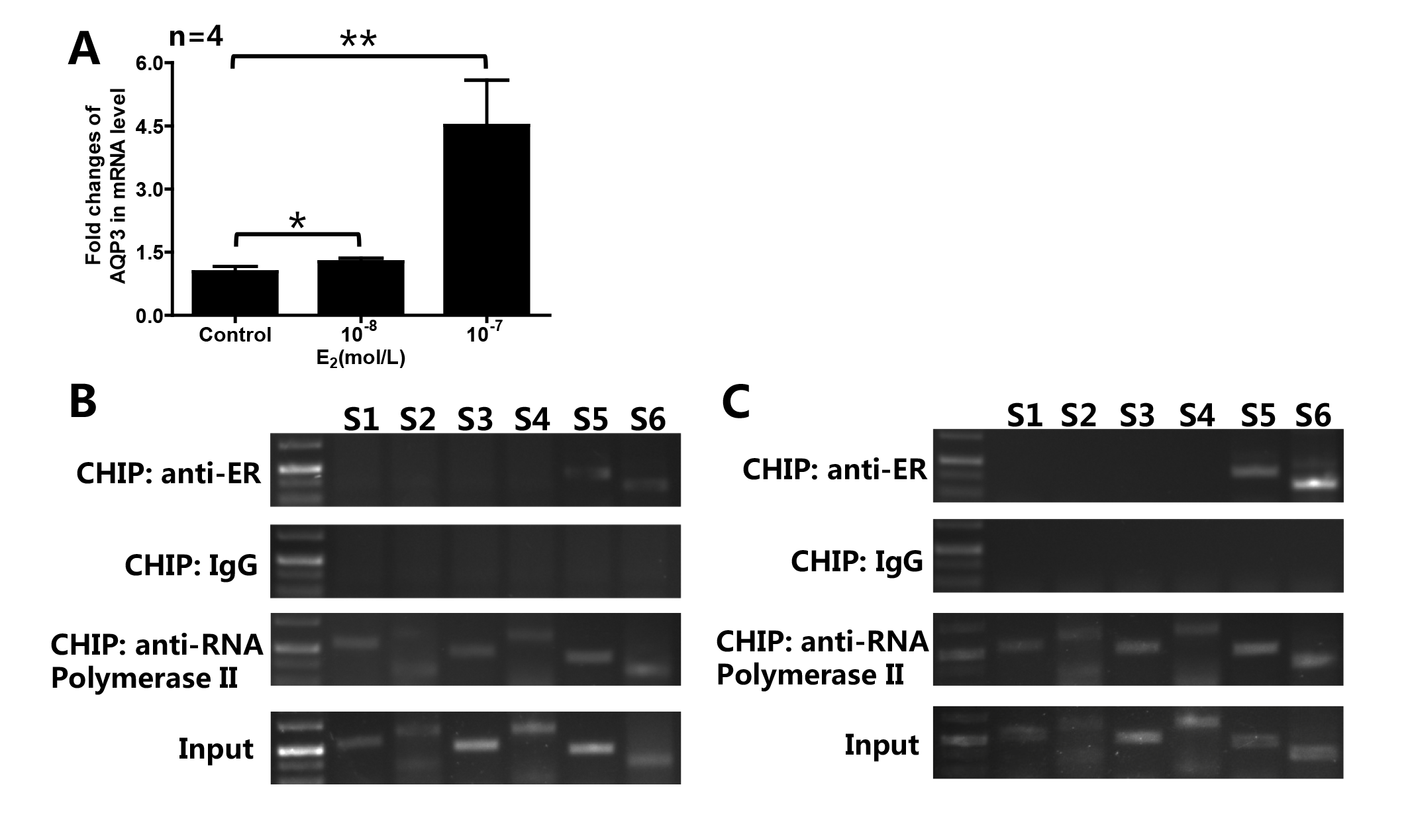


**Supplementary Figure S2: E_2_ upregulated AQP3 expression in MCF7 cells.** **(A)** E_2_ upregulated expression of AQP3 in mRNA level. **(B** and **C)** ChIP analysis included positive control (anti-RNA polymerase II), negative control (normal mouse IgG), ERα antibody and input groups. Two sequences (S5 and S6) were pulled down by anti-ERα antibody, and were brighter in the presence of E_2_ **(C)** than in the absence of E_2_ **(B)**. The data are presented as mean ± SD. **P*<0.05 and ***P*<0.01 vs. Control (One-way ANOVA and Turkey’s post hoc tests).


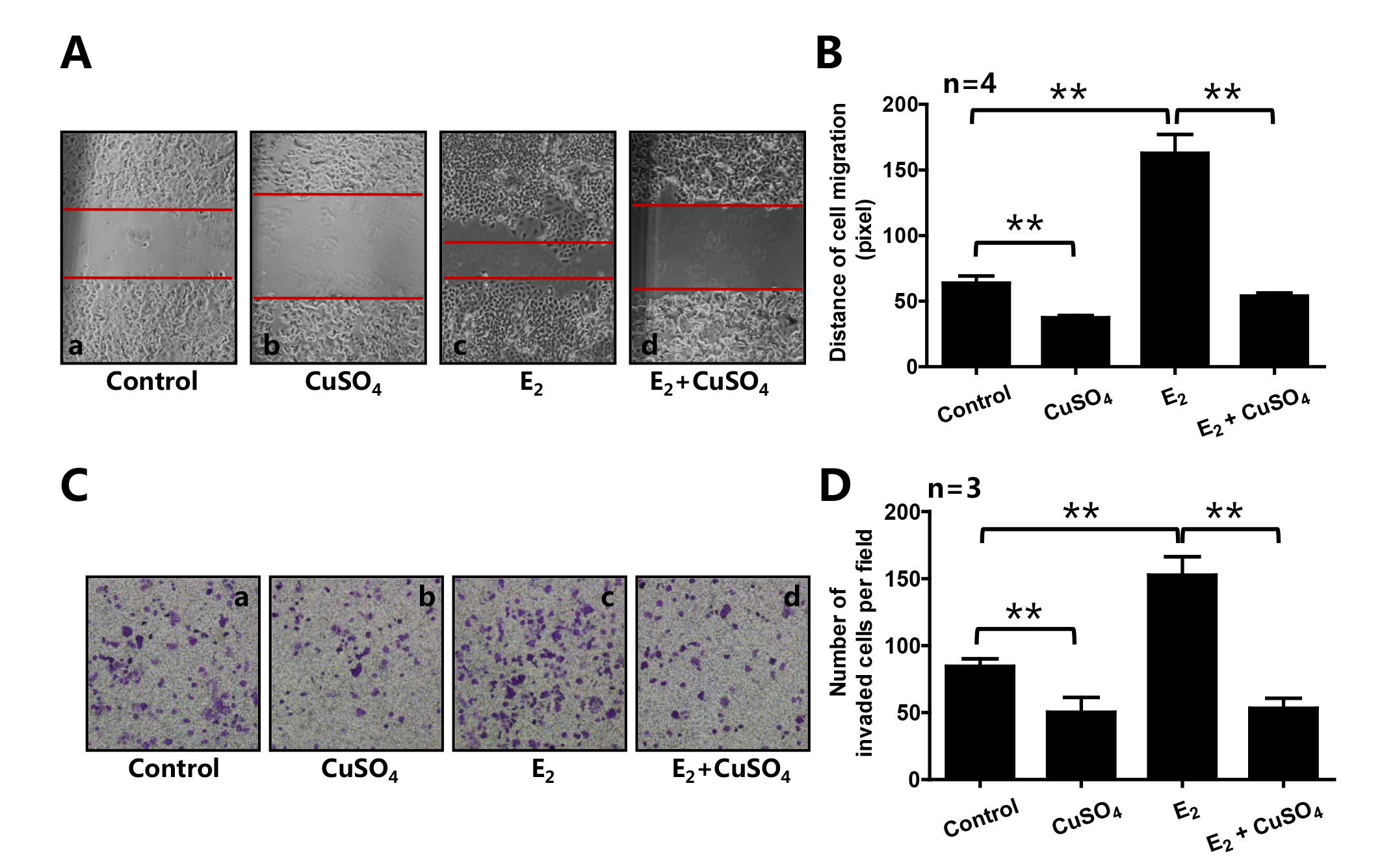


**Supplementary Figure S3: CuSO_4_ reduced E_2_-induced cell migration and invasion of T47D cells.** T47D cells were treated with or without E_2_ (10^-7^ M) and CuSO_4_ (200 μM). E_2_ significantly increased cell migration **(Ac** and **B)** and invasion **(Cc** and **D)**, and, CuSO_4_ treatment significantly attenuated E_2_-promoted migration **(Ad** and **B)** and invasion **(Cd** and **D)** of T47D cells. CuSO_4_ treatment also attenuated migration **(Ab** and **B)** and invasion **(Cb** and **D)** of T47D cells in the absence of E_2_. The data are presented as mean ± SD, ***P*<0.01 (One-way ANOVA and Turkey’s post hoc tests).


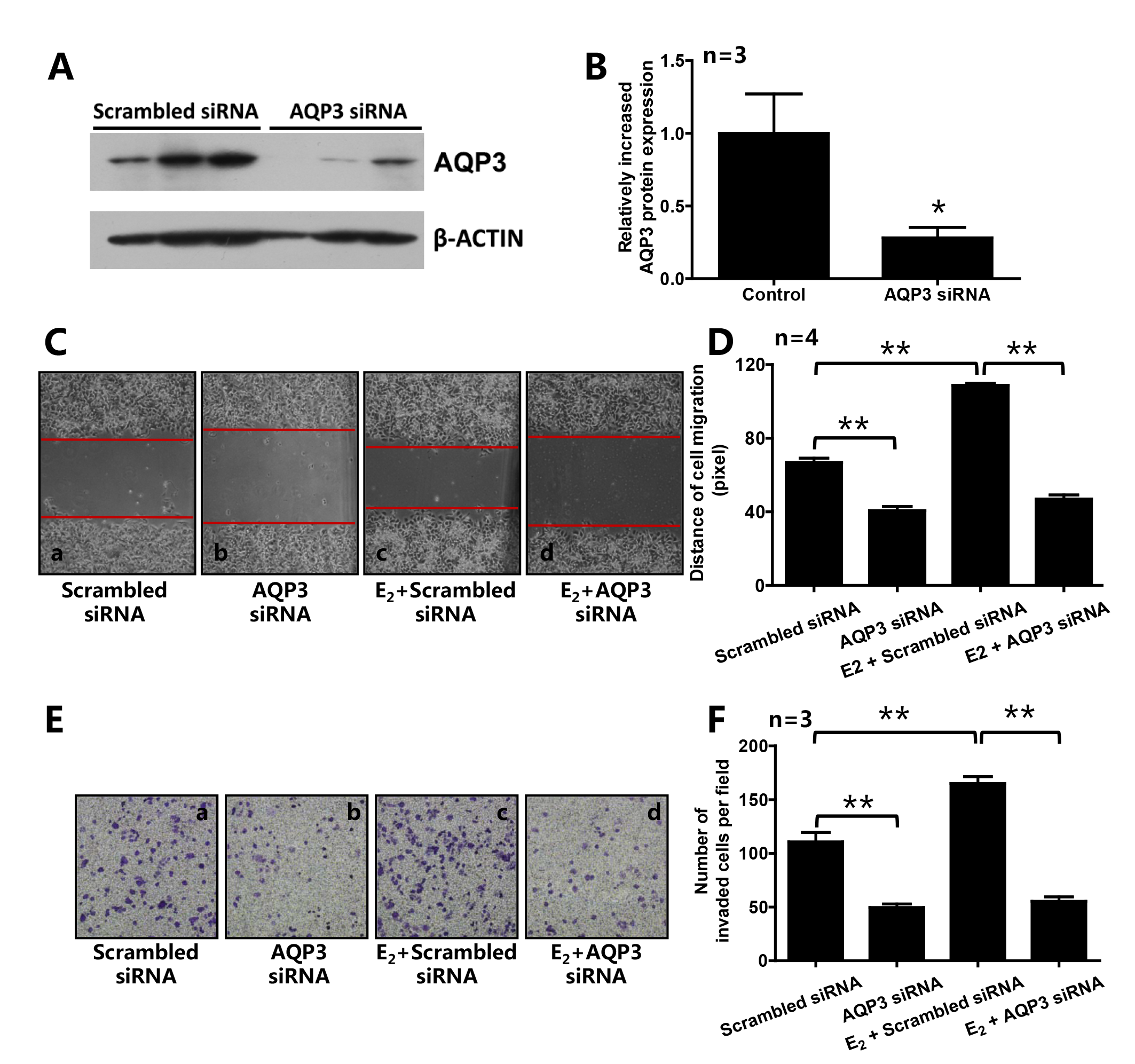


**Supplementary Figure S4: Knockdown of AQP3 reduced E_2_-induced cell migration and invasion of MCF7 cells.** **(A** and **B)** Treatment of MCF7 cells with AQP3-specific siRNA significantly reduced the expression level of AQP3. E_2_ (10^-7^ M) significantly increased cell migration **(Cc** and **D)** and invasion **(Ec** and **F)** in scrambled siRNA group, and, knockdown of AQP3 in MCF7 cells inhibited the E_2_-promoted migration **(Cd** and **D)** and invasion **(Cd** and **D)**. Knockdown of AQP3 also attenuated migration **(Cb** and **D)** and invasion **(Eb** and **F)** of MCF7 cells in the absence of E_2_. The data are presented as mean ± SD, ***P*<0.01 (B: Student *t*-test; D and F: One-way ANOVA and Turkey’s post hoc tests).

| **Supplemental Table S1. Primer sequences used for ChIP analysis of *AQP3* gene**. | | | | | | |
| --- | --- | --- | --- | --- | --- | --- |
| **Sequences containing Putative ERE** | **ERE Position** | | **Putative ERE sequence** | **RAST score** | **Primer sequence** | **Product size** |
|  | **Start** | **End** |  |  |  |  |
| **S1** | -4540 | -4522 | TGAGGTTGCAGTGAGCAGA | 7.3 | F- CAGCCCACGAGAAAACCCAT | 217 |
|  |  |  |  |  | R- CATGGTGGTAGGCACCTGTA |  |
| **S2** | -2652 | -2634 | AGAGGTTGCAGTGAGCCAA | 8.5 | F- GAAGCAGGAGAATCGCTTG | 146 |
|  |  |  |  |  | R- TCTCTTTGGGCATCAACCAC |  |
| **S3** | -1965 | -1947 | GGAAGTTGCAGTGACCTGA | 9.9 | F- AGCCCTGCTCGCTTCTCAATGA | 196 |
|  |  |  |  |  | R- CGTGATGGCGGGTGCCTGTAAT |  |
| **S4** | -1829 | -1811 | CAAGACCACACTGACCAAC | 7.9 | F- GTTTGGCTCTTGTTGCCCAG | 250 |
|  |  |  |  |  | R- AATCCCAGCACTTTGGGAGG |  |
| **S5** | -1340 | -1322 | CGAGGCTACAGTGAGCTGT | 7.4 | F- GTCCCATCCATTCTGGAGGC | 178 |
|  |  |  |  |  | R- ATTATGGGGGAGTCAGCCCT |  |
|  |  |  |  |  | F- CGG**GGTACC**GTCCCATCCATTCTGGAGGC |  |
|  |  |  |  |  | R- CCG**CTCGAG**ATTATGGGGGAGTCAGCCCT |  |
| **S6** | -731 | -713 | ACATGGCTAGGTGACCTAG | 8.2 | F- TCAGCCTCTGGAGCCATGTGAG | 134 |
|  |  |  |  |  | R- GCGGATGTCCTGTGTGCCTAAT |  |
|  |  |  |  |  | F- CGG**GGTACC**TCAGCCTCTGGAGCCATGTGAG |  |
|  |  |  |  |  | R- CCG**CTCGAG**GCGGATGTCCTGTGTGCCTAAT |  |

| **Supplemental Table S2. Primer sequences used for qPCR.** | | | |
| --- | --- | --- | --- |
| **Gene** | **Primer sequence** | **Product size** | **Protein** |
| *AQP3* | F- GAGATGCTCCACATCCGCTA | 88 | AQP3 |
|  | R- AGCCACAGCCAAACATCACC |  |  |
| *GAPDH* | F-CAGGGCTGCTTTTAACTCTGG | 102 | GAPDH |
|  | R- TGGGTGGAATCATATTGGAACA |  |  |
| *CDH1* | F-AAGCCCCCATCTTTGTGCCTCCT | 157 | E-cadherin |
|  | R-TCCAGCCAGTTGGCAGTGTCTCT |  |  |
| *TJP1* | F-GCACAGTTTGGCACAGCCTCCT | 210 | ZO-1 |
|  | R-AGCACGCCCCCATTGCTGTT |  |  |
| *CTNNB1* | F-TTGCGTGAGCAGGGTGCCATT | 157 | β-catenin |
|  | R-TGTGAAGGGCTCCGGTACAACCT |  |  |
| *VIM* | F-CCAAGAACCTGCAGGAGGCAGAAG | 192 | Vimentin |
|  | R-GCATCTGGCGTTCCAGGGACTCAT |  |  |
| *FN1* | F-AGTGCATCTGCACAGGCAACGG | 201 | Fibronectin 1 |
|  | R-TCAGCCACTGCATCCCCACAGA |  |  |
| *CDH2* | F-TGACTCCAACGGGGACTGCACA | 154 | N-cadherin |
|  | R-CTGGCGTTCTTTATCCCGGCGT |  |  |
| *SNAI2* | F-TGCGGCAAGGCGTTTTCCAGA | 239 | Slug |
|  | R-CAGTGTGCTACACAGCAGCCAGA |  |  |
| *SNAI1* | F-TACAAGGCCATGTCCGGACCCA | 174 | Snail |
|  | R-TGTGGAGCAGGGACATTCGGGA |  |  |

**
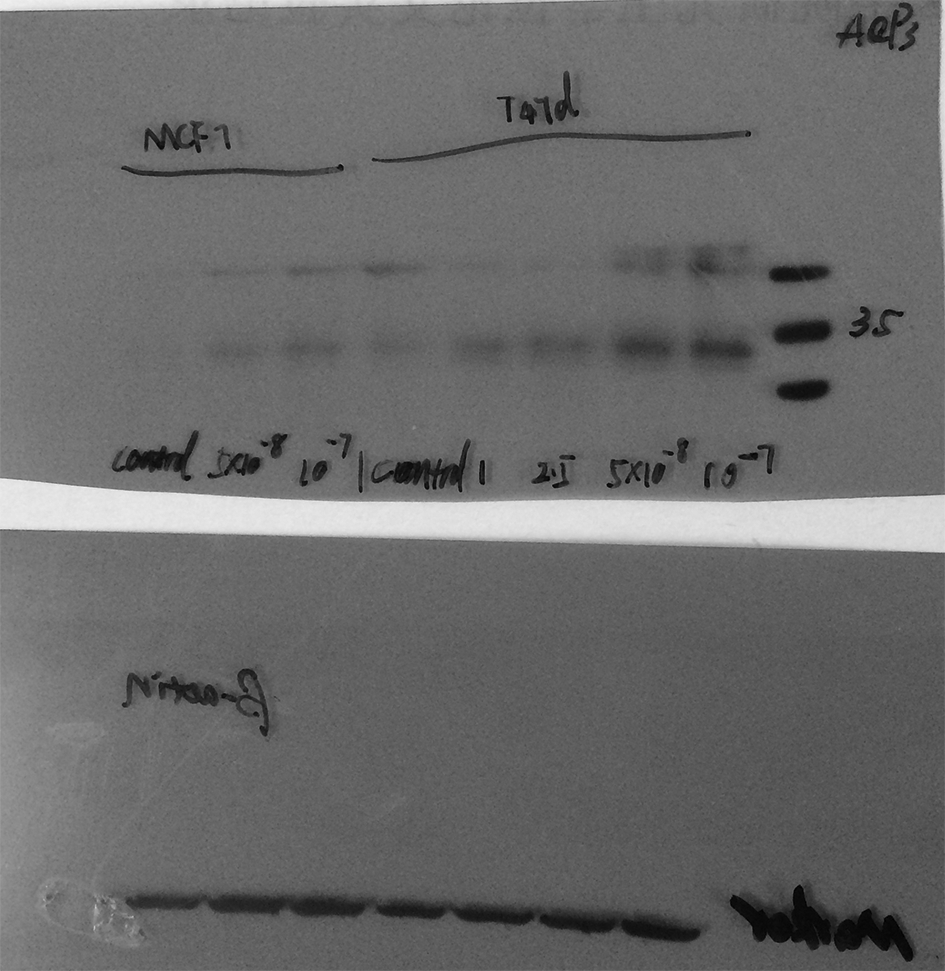
**

**Supplementary Figure S5: Full gel run of Figure 2E.**

**
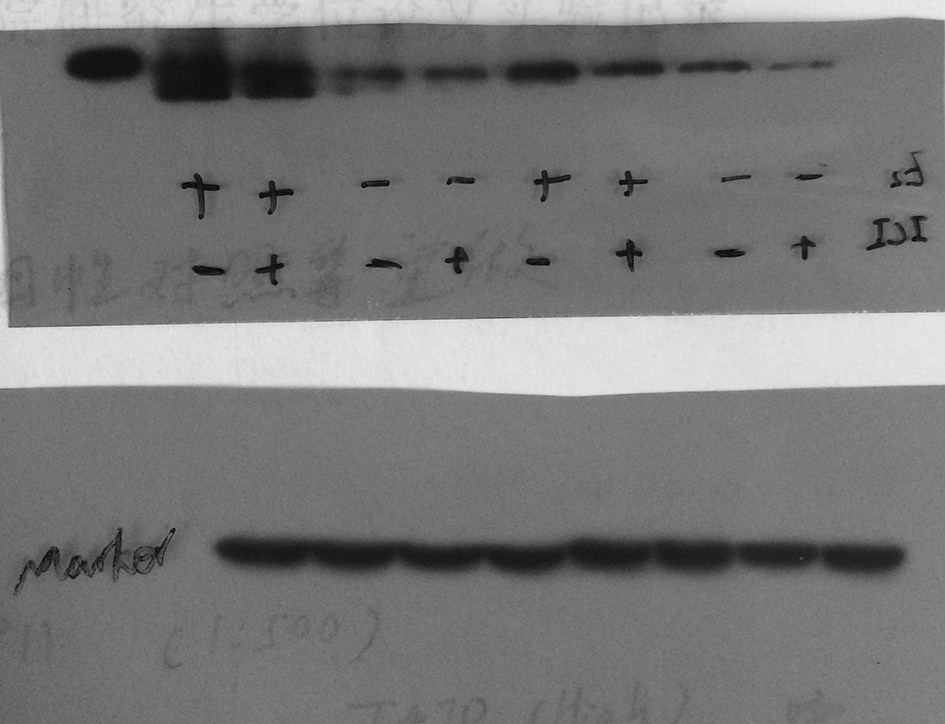
**

**Supplementary Figure S6: Full gel run of Figure 2G.**

**
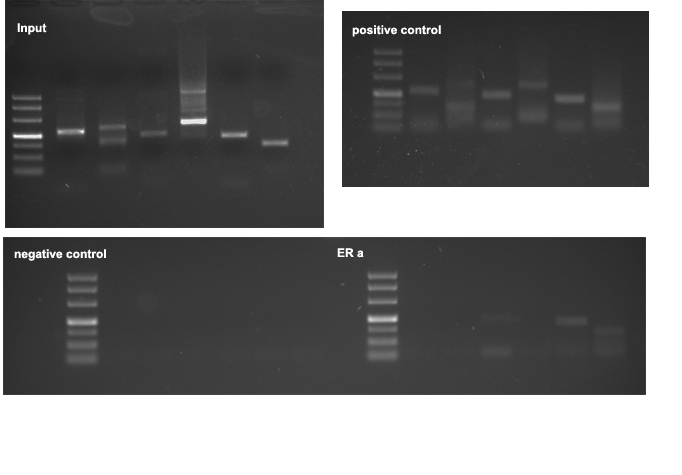
**

**Supplementary Figure S7: Full gel run of Figure 3B.**

**
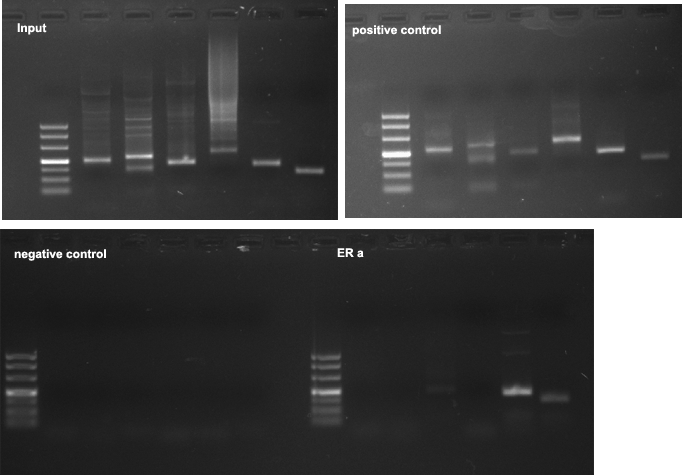
 Supplementary Figure S8: Full gel run of Figure 3C.**

**
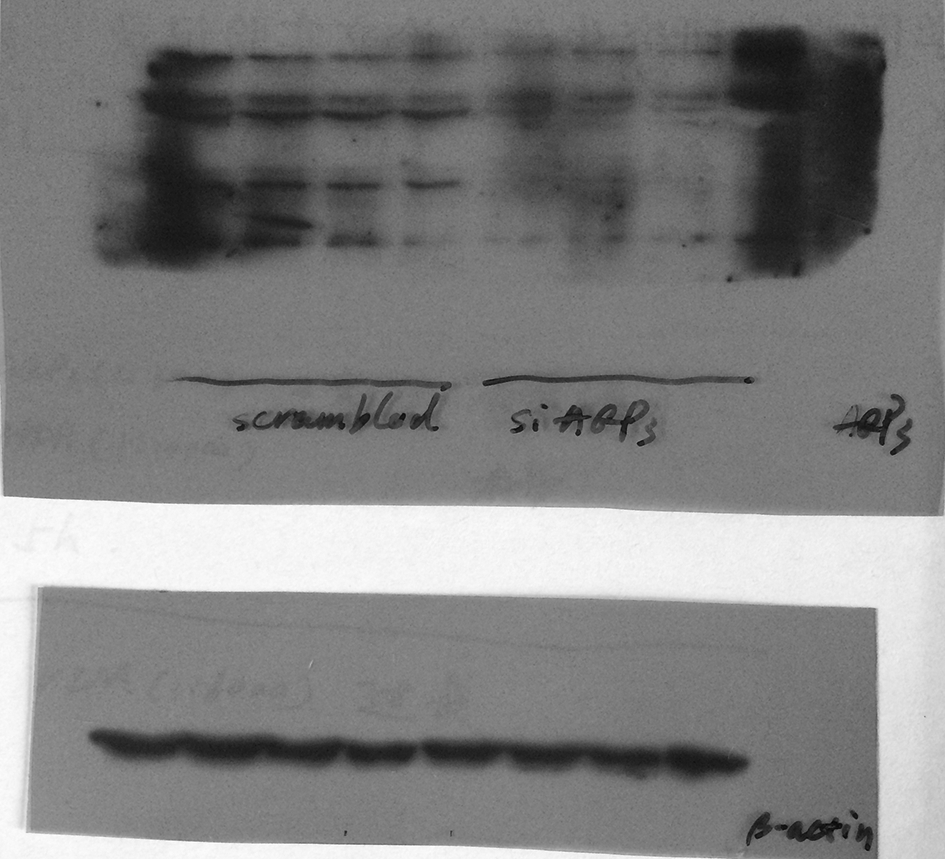
**

**Supplementary Figure S9: Full gel run of Figure 4A.**

**
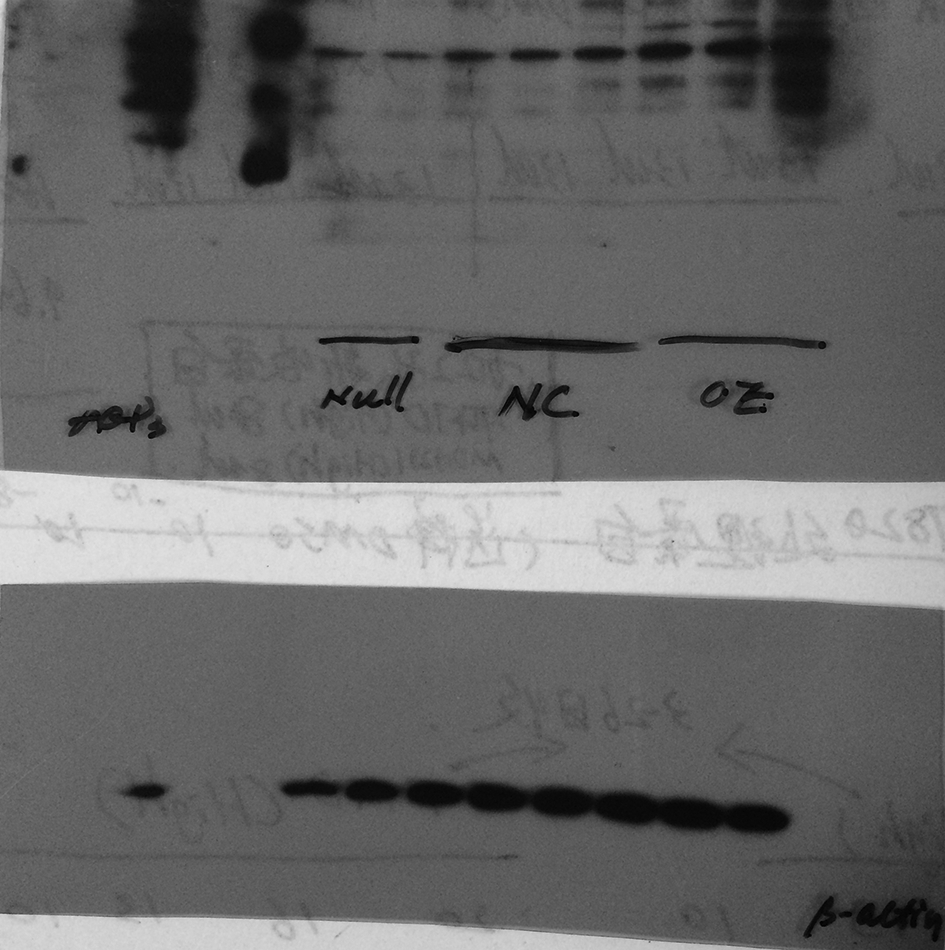
**

**Supplementary Figure S10: Full gel run of Figure 5A.**

**
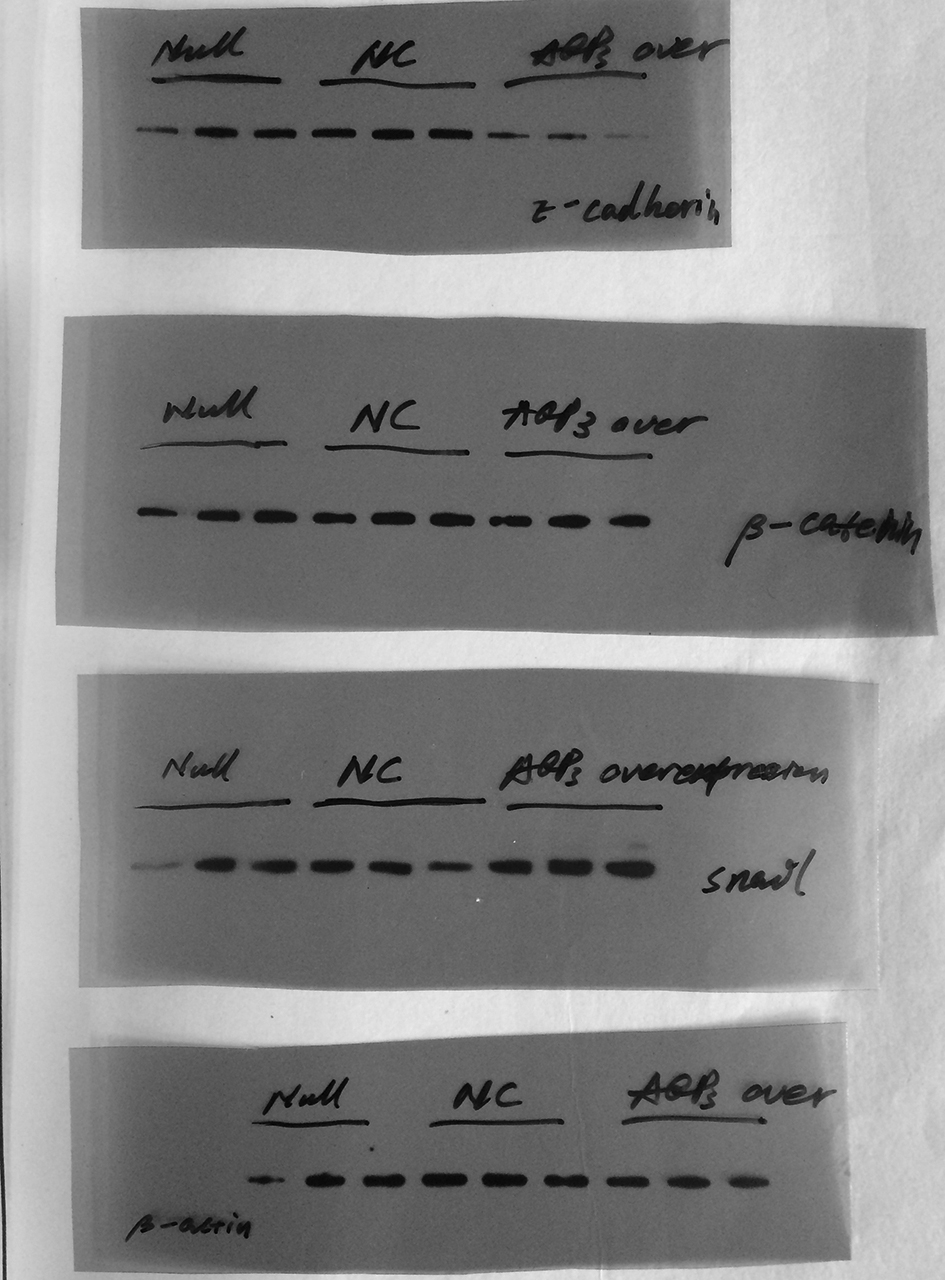
**

**Supplementary Figure S11: Full gel run of Figure 6E.**

**
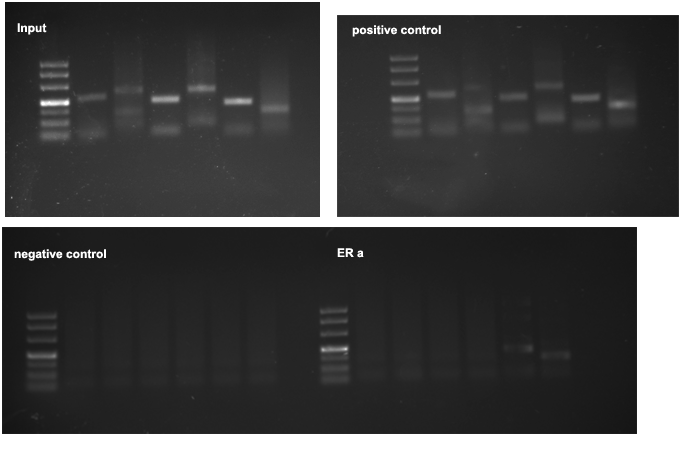
Supplementary Figure S12: Full gel run of Supplementary Figure S2B.**

**
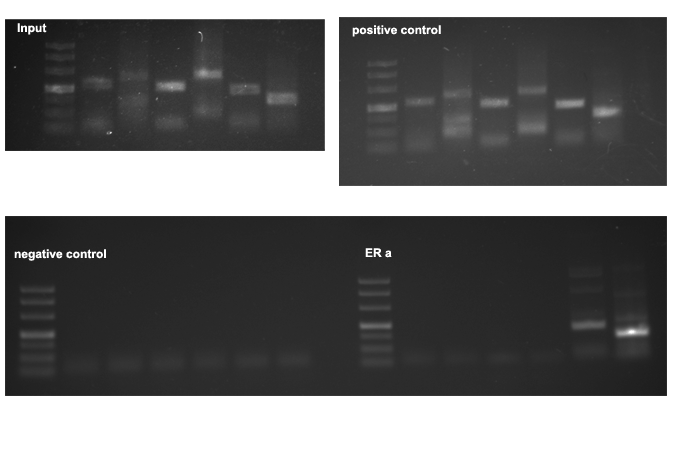
**

**Supplementary Figure S13: Full gel run of Supplementary Figure S2C.**

**
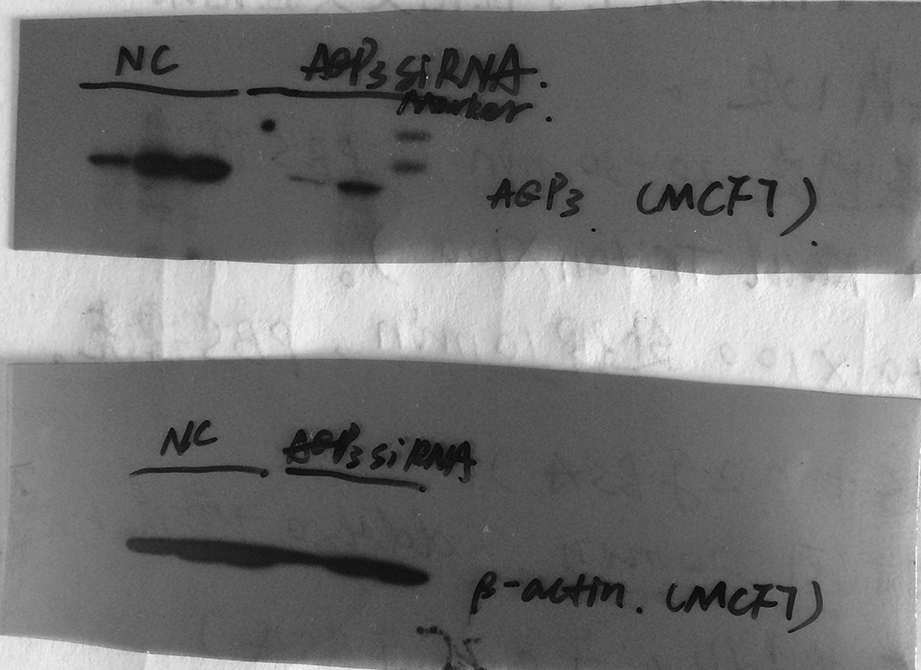
**

**Supplementary Figure S14: Full gel run of Supplementary Figure S4A.**
